# Measurement of superficial and deep abdominal muscle thickness: an ultrasonography study

**DOI:** 10.1186/s40101-016-0106-6

**Published:** 2016-08-23

**Authors:** Nahid Tahan, Khosro Khademi-Kalantari, Mohammad Ali Mohseni-Bandpei, Saeed Mikaili, Alireza Akbarzadeh Baghban, Shapour Jaberzadeh

**Affiliations:** 1 Physiotherapy Research Center, School of Rehabilitation, Shahid Beheshti University of Medical Sciences, Tehran, Iran; 2 Department of Physiotherapy, School of Rehabilitation, Shahid Beheshti University of Medical Sciences, Tehran, Iran; 3 Iranian Research Centre on Aging, Department of Physiotherapy, University of Social Welfare and Rehabilitation Sciences, Evin, Tehran Iran; 4 Faculty of Medicine, Nursing and Health Sciences, School of Primary Health Care, Monash University, Melbourne, Australia

## Abstract

**Background:**

Real-time ultrasound imaging is a valid method in the field of rehabilitation. The ultrasound imaging allows direct visualization for real-time study of the muscles as they contract over the time. Measuring of the size of each abdominal muscle in relation to the others provides useful information about the differences in structure, as well as data on trunk muscle activation patterns. The purpose of this study was to assess the size and symmetry of the abdominal muscles at rest in healthy adults and to provide a reference range of absolute abdominal muscle size in a relatively large population.

**Method:**

A total 156 healthy subjects with the age range of 18–44 years were randomly recruited. The thickness of internal oblique, external oblique, transverse abdominis, and rectus abdominis muscles was measured at rest on both right and left sides using ultrasound. Independent *t* test was used to compare the mean thickness of each abdominal muscle between males and females. Differences on side-to-side thicknesses were assessed using paired *t* test. The association between abdominal muscle thicknesses with gender and anthropometric variables was examined using the Pearson correlation coefficient.

**Results:**

A normal pattern of increasing order of mean abdominal muscle thickness was found in both genders at both right and left sides: transverse abdominis < external oblique < internal oblique < rectus abdominis. There was a significant difference on the size of transverse abdominis, internal oblique, and external oblique muscles between right and left sides in both genders. Males had significantly thicker abdominal muscles than females. Age was significantly correlated with the thickness of internal oblique, external oblique, and rectus abdominis muscles. Body mass index was also positively correlated with muscle thickness of rectus abdominis and external oblique.

**Conclusions:**

The results provide a normal reference range for the abdominal muscles in healthy subjects and may be used as an index to find out abnormalities and also to evaluate the effectiveness of different interventions.

The lateral abdominal muscles including transversus abdominis (TrA), internal oblique (IO), and external oblique (EO) provide stability to the trunk in different functional activities [1, 2]. The assessment of the size and thickness of abdominal muscles is important for the management of patients with low back pain (LBP) and during athletic training [3–5]. Researchers have developed normative reference ranges for abdominal size and symmetry to help identify potential muscle aberrations in various age groups in both genders [6–8]. There are different imaging techniques for the assessment and evaluation of the muscle thickness. This include magnetic resonance imaging (MRI) [3, 9] and computerized tomography scanning (CTS) [10, 11]; however, these methods are very expensive which make them unsuitable especially for large scale studies. Recently, there is a growing interest in the use of real-time ultrasound imaging (UI) as a valid method in the field of rehabilitation [12–14] as well as to evaluate abdominal muscle structure and function in studies on healthy individuals [2, 4, 15, 16]. Some studies support higher reliability and validity of real-time UI in the measurement of muscle geometry compared to other well-accepted techniques such as magnetic resonance imaging (MRI) and electromyography (EMG) [3, 17].

Measurement of abdominal muscle thickness in different positions and health conditions provides useful information about the structural changes in muscle structure that can be attributed to the related positions or conditions. In addition, comparison of each abdominal muscle thickness with other muscles in the same side or the muscles on opposite side may help to determine whether there is a consistent order and relative thickness, which could be used as a guide for the assessment of imbalance within the abdominal muscle groups in both younger and older healthy adults. Normal muscle values may vary in different societies which could be affected by culture, nutritional status, and physical activities. As there is no published evidence available on normal values of abdominal muscles in Iranian population, therefore, the present study was designed (a) to determine the thickness and symmetry of the abdominal muscles at rest in a relatively large population of healthy Iranian adults and (b) to investigate the associations between some demographic factors such as gender, age, and body mass index (BMI) and abdominal muscle thickness in healthy individuals.

## Materials and methods

A total of 156 healthy volunteers (75 males, 81 females) with age range of 18-44 years, participated in this study. The age, height, and weight of participants (mean ± standard deviation) were 24.3 ± 7.2 years, 167.6 ± 8.9 cm, and 65.3 ± 11.9 kg, respectively.

Participants were evaluated by a physician to rule out any pain or dysfunction in their lower back, pelvis, thoracic, lower extremities, or any previous surgery involving abdominal muscles which may affect their size and function. Participants were excluded if they had any history of neuromuscular, musculoskeletal, cardiopulmonary, or inflammatory diseases.

The study followed the Declaration of Helsinki and was approved by the Human Ethics Committee at Shahid Beheshti University of Medical Sciences, Tehran, Iran. All participants were given a written explanatory statement about the procedure and risks involved in this study, and then they were asked to sign a consent form if they were willing to take part in the study.

**Fig. 1:**
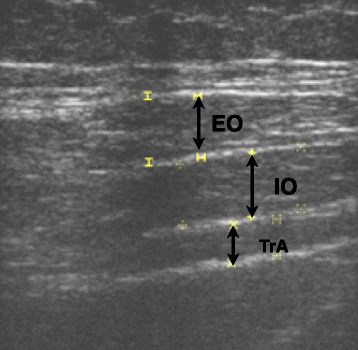
An ultrasound imaging of the lateral abdominal wall muscles taken during resting state. *TrA* transversus abdominis, *IO* internal oblique, *EO* external oblique

The ultrasonography device used in this study was an imaging unit set in B mode (HS -2100 Honda Electronic) with 7.5-MHz linear array transducer. Thickness of the TrA, IO, EO, and rectus abdominis (RA) muscles has been measured by an experienced and qualified physiotherapist using UI. Abdominal muscle thicknesses were measured at rest on both right and left sides. Participants were positioned in supine crook-lying while pillows were placed under their head and knees [18]. The angle of knees was checked by a hand goniometer, and the position of lumbar spine was assessed visually. The abdominal wall was exposed, and the inferior border of the rib cage and iliac crest was marked as reference points. Ultrasound gel was used between the transducer and the skin to increase the area of contact and to minimize the need for inadequate inward probe pressure [19]. For antero-lateral abdominal wall muscles (TrA, IO, EO), the UI transducer was transversely located across the right side of the abdominal wall over the anterior axillary line, midway between the 12th rib and the iliac crest, to obtain a clear image of the deep abdominal layers. For anterior abdominal wall muscle (RA), the transducer was placed 2–3 cm above the umbilicus, 2–3 cm from the midline. All images were captured directly at the end of the expiration, as determined by the visual inspection of the abdominal content. Two images of each muscle were taken at rest and the mean of the two measurements were used in the statistical analyses. Typical images of the lateral abdominal muscles at rest are shown in Fig. 1.

Test-retest reliability of the ultrasound measurements of all abdominal muscle was assessed in a pilot study on 10 healthy participants. Measurements in each participant were taken twice 30 min apart.

### Data analyses

One-sample Kolmogrov-Smirnov test was used to assess the normality of distribution for the UI measurement of muscle thickness in all abdominal muscle at rest. Normal distribution was observed for variables. The reliability data were analyzed using intra class correlations (ICCs). We used a three-way ANOVA (2 genders × 4 muscles × 2 sides) to compare thickness of different abdominal muscles in both male and female participants in each side and also with contralateral side of the body. If required, pairwise comparisons with the Bonferroni correction (using independent *t* test) were used to identify where the differences were significant. The association between abdominal muscle thicknesses with gender, age, and BMI was examined using Pearson’s correlation coefficient and multiple regression analysis. The analysis was performed using SPSS software version 20, and statistical level of significance was set at 0.05.

## Results

The results of the muscle thickness measurement in this study showed that the ICC for the test–retest reliability for the sonographic measurement of all four muscle thicknesses were very high (0.89–0.96). Descriptive statistics of different abdominal muscle thickness in both males and females are provided in Table 1. We used ANOVA to compare thickness of different abdominal muscles in both male and female participants in each side and also with contralateral side of the body. The main effect of muscle was significant (*P* = 0.001). Post hoc analysis with the Bonferoni correction showed that the differences between the all six comparisons were also significant (*P* = 0.001) (Table 2). This data also indicates a normal pattern of increasing order of mean resting abdominal muscle thickness bilaterally without considering participants gender and BMI in the analysis: TrA < EO < IO < RA. Analysis also revealed that males had significantly thicker abdominal muscles than females (*P* < 0.001). In addition, a significant difference was found between right and left side measurements in all muscles except for RA (regardless of the gender): *P* < 0.001 for TrA, *P* < 0.001 for IO, and *P* < 0.01 for EO (Table 3). The side-to-side difference for RA muscle was not statistically significant (*P* = 0.16).

**Table 1:** The averaged ultrasound thickness measurements (in mm) in each gender

Muscle	Gender	Mean	SD	Minimum	Maximum
TrA (Rt)	Men	4.5	0.9	2.3	7
	Women	3.5	0.8	2	6.9
TrA (Lt)	Men	3.8	1	1.9	6.9
	Women	3.3	0.7	1.9	5.6
IO (Rt)	Men	8.9	2.3	3.3	14.5
	Women	6.1	1.3	3.9	11.2
IO (Lt)	Men	8.5	2	2.8	14
	Women	5.8	1.2	3.4	9.7
EO (Rt)	Men	5.7	1.2	3.7	9.2
	Women	4.8	1.1	2.6	8.8
EO (Lt)	Men	5.4	1.3	2.2	8.3
	Women	4.8	1.1	2.6	8.8
RA (Rt)	Men	10.3	1.8	7	16.2
	Women	8.7	1.2	6.5	12.1
RA (Lt)	Men	10.4	1.9	6.7	17
	Women	8.3	1.3	5.7	12

*SD* standard deviation, *TrA* transversus abdominis, *IO* internal oblique, *EO* external oblique, *RA* rectus abdominis, *(Rt)* right, *(Lt)* left

**Table 2:** Pair wise thickness comparisons based on the Bonferroni test

(I) muscle	(J) muscle	Mean difference (I–J)	Std. error	Sig
	IO	−3.39	.133	.000
TrA	EO	−1.19	.078	.000
	RA	−5.49	.128	.000
	EO	2.19	.136	.000
IO	RA	−2.10	.153	.000
EO	RA	−4.29	.112	.000

**Table 3:** A comparison of side-to-side muscle thickness (mm) differences

Muscle	Mean	SD	t	Sig. (two-tailed)
TrA (Rt)–TrA (Lt)	0.20	.79	3.22	0.002
IO (Rt)–IO (Lt)	0.40	1.57	3.22	0.002
EO (Rt)–EO (Lt)	0.26	1.24	2.62	0.010
RA (Rt)–RA (Lt)	0.13	1.19	1.4	0.167

*TrA* transversus abdominis, *IO* internal oblique, *EO* external oblique, RA rectus abdominis, *(Rt)* Right, *(Lt)*: left, *SD* standard deviation

Using multiple regression analysis, age showed a statistically significant negative interaction with thickness of IO, EO, and RA muscles (Table 4). In addition, the result of the Pearson correlation coefficient demonstrated that there was a statistically significant association between gender and muscle size (*P* < 0.05 for all muscles). Age showed significant negative correlation with IO (*r* = −0.182, *P* = 0.023), EO (*r* = −0.197, *P* = 0.014), and RA (*r* = −0.214, *P* = 0.002) thickness, but the association was not statistically significant for TrA (*P* > 0.05). BMI was positively associated with the thickness of RA muscle (*P* < 0.001) and EO (*P* < 0.01), but no significant correlation was found between BMI and thickness of TrA or IO muscles (*P* > 0.05).

**Table 4:** Multiple regression analysis to evaluate association between age and abdominal muscle thickness after adjusting for sex and BMI

Dependent variable (muscle thickness)	*B* (coefficient)	Std-error	*t*	*P* value
IO	−0.04	0.02	−2.17	0.03
EO	−0.04	0.01	−2.88	0.00
RA	−0.07	0.01	−3.74	.000

*IO* internal oblique, *EO* external oblique, *RA* rectus abdominis

## Discussion

In this study, a normative data on abdominal muscle thickness from a relatively large sample of Iranian healthy participants is provided. Regardless of the gender, in both male and female group, the difference in the abdominal muscle thickness demonstrated similar pattern reported in previous studies (i.e., RA appeared to be the thickest muscle followed by IO, EO, and TrA). This finding is consistent with the results of previous studies [6, 8, 20]. Any abnormal pattern of abdominal muscle thickness in any case may be attributed to the existence of muscle atrophy in specific muscle. In addition, the findings of present study can also be used as normative values to assess postural abnormalities and possible muscle imbalance in various pathologies.

The result of current study reveals a negative correlation between age and the muscle thickness in all investigated muscles except for TrA. Significant reduction in the thickness of IO, EO, and RA was observed through aging. The thickness of TrA was not found to be significantly correlated with age, and this was not in agreement with the findings of Rankin et al. [7], although they reported a poor correlation (*r* = 0.42) between TrA and age.

The result of the present study is consistent with the findings of Ota et al. [21]. They reported that the thickness of abdominal muscles has a significant negative linear correlation with age. They also demonstrated that this correlation was observed in all abdominal muscles except for TrA. However, the results suggest that changes in muscular thickness with aging can mainly affect the more superficial abdominal muscles rather than deep muscles such as TrA. This finding can be used in rehabilitation of abdominal muscles in patients with LBP.

The main role of deep abdominal muscle such as transverse abdominis is to maintain the stability of the lumbar spine against the effects of gravity during daily physical activities [22]. To do this, the deep muscle use only very low level of contraction about 2 to 3 % of their maximum voluntary contraction level [23]. Disregarding the age, as far as we are involved in the activities in upright positions, the deep abdominal muscles are active to keep the lumbar spine in its neutral position. The result of the present study suggests that the mass of transverse abdominis muscle may be maintained by this small amount of contraction regardless of the age.

There are differences in the composition of the deep compared to superficial abdominal muscles that can explain their different response to the aging process [24, 25]. TrA muscle as lumbar stabilizer is mainly composed of type I fiber compared to RA that is mainly consisted of type II fiber [26]. It is shown that the type II fibers will show greater shrink in size compare to type I fiber when they are opposed to atrophic situations although, the loss of actual fibers themselves is similar for type I and type II [27]. This could explain the significant negative correlation between superficial muscle size and the age and the not significant correlation for the deep muscles we found in our study.

Although a significant correlation was found between the thickness of EO and RA muscles and BMI of the participants, no significant correlation was found between the thickness of TrA and IO muscles and BMI. It seems that individuals with higher BMI had thicker superficial layers of abdominal muscles compared to deep layers. In a study conducted by Springer et al. [8], a positive significant relationship between the BMI and thickness in all muscles of external abdominal layer was reported in 32 participants.

One of the limitations of this research was measuring muscle thickness instead of muscle cross-sectional area as a measure of muscle size of the abdominal muscles. Another limitation was related to the small sample of left-handed subjects. In the present study, only nine subjects were left handed; therefore, it was not possible to determine the effect of hand dominancy on muscle thickness.

Results from normative values of the abdominal muscle thickness presented in this study may assist clinicians and researchers to investigate muscle imbalance in pathological conditions especially in patients with LBP and also can be used as an index to evaluate the effectiveness of any interventions.

## Conclusions

The results of this study demonstrated a normal pattern of increasing order of mean abdominal muscle thickness emerged in both genders at both sides of body: TrA < EO < IO < RA. In addition, the results indicated a significant difference in TrA, IO, and EO muscle thickness between right and left sides in both genders. Males seemed to have significantly thicker abdominal muscles than females. Age was significantly correlated with IO, EO, and RA muscle thickness and also BMI was positively associated with muscle thickness in RA and EO.
